# Knowledge of Iranian Parents of Elementary School Children about Traumatic Dental Injuries and its Management

**DOI:** 10.30476/DENTJODS.2020.84483.1085

**Published:** 2020-09

**Authors:** Fatemeh Kebriaei, Hajar Attarzadeh, Leyli Sadri, Elnaz Foroughi, Mehdi Taghian, Soroush Sadri

**Affiliations:** 1 Dept. of Pediatric Dentistry, School of Dentistry, Shahid Sadoughi University of Medical Sciences, Yazd, Iran; 2 Dept. of Pediatric Dentistry, School of Dentistry, Mazandaran University of Medical Sciences, Sari, Mazandaran, Iran; 3 Dept. of Pediatric Dentistry, School of Dentistry, Arak University of Medical Sciences, Arak, Iran; 4 Dept. of Oral and Maxillofacial Surgery, School of Dentistry, Mazandaran University of Medical Sciences, Sari, Mazandaran,Iran; 5 Medical Students' Research Center, Isfahan University of Medical Sciences, Isfahan, Iran

**Keywords:** Dental trauma, Avulsed teeth, Knowledge

## Abstract

**Statement of the Probelm:**

Traumatic dental injuries (TDIs) are frequent events during childhood, and emergency management of these injuries has positive outcomes.

**Purpose:**

The aim of this study was to evaluate the knowledge of parents of school-aged children about dental trauma and to identify the effect of demographic variables such as age, gender, and education on their responses.

**Materials and Method:**

300 parents of elementary school children were selected through cluster sampling from July 2012 to January 2013 in Yazd, Iran. A questionnaire was designed to collect the data on parents’ knowledge about emergency management of dental trauma, and their demographic characteristics and previous experiences. Statistical analysis of data was performed by SPSS software version 11 using T-test and ANOVA. P-values less than 0.05 were considered significant.

**Results:**

296 out of 300 participants returned their questionnaires (mean age 33.8± QUOTE ± 5). The parents’ knowledge about TDI managements was inadequate (average score = 7.03). According to T-test and ANOVA statistical tests, there was no significant relationship between knowledge and age (*p*= 0.155), gender of parents (*p*= 0.113), gender of children (*p*= 0.776), occupation (*p*= 0.112), and the information source (*p*= 0.160). The relationship between parents’ knowledge and parental educational level was statistically significant (*p*= 0.010), and least significant difference (LSD) test showed that knowledge score of parents with Bachelor’s degree or higher educational levels (7.83±4) was significantly more than other parents who were not educated (6.97±4), or had high school diploma (6.70±4).

**Conclusion:**

Majority of parents had little knowledge about TDI and emergency management of avulsed permanent teeth in children. Therefore, it seems that educational programs are necessary to improve parents’ knowledge.

## Introduction

Today, dental traumas are considered an important concern among dental problems in human [ 1 ]. American Society of Oral surgeons described the management of traumatic dental injuries (TDIs) as one the most sensitive emergencies [ 2 , 3 ], and based on the literature, these injuries has increased in recent decades [ 4 ]. One of the most considerable adverse outcomes of TDI is its negative effect on the child’s quality of life. Furthermore, the costs imposed by these traumatic injuries are not negligible [ 5 ]. It is also shown that 16% of TDIs lead to negative changes in appearance and development of children’s face [ 6 ]. The prognosis and success of TDIs’ treatment strongly relate to an immediate and proper reaction that depends on many factors including parental awareness especially mothers [ 7 ]. In conclusion, parental awareness of reasons and management of TDIs including first aid measures, proper storage media, and tetanus vaccine can be of critical significance for saving teeth as well as maintaining the child’s general health.

TDIs are very common in childhood. It is shown that one third of preschool children and one fourth of schoolchildren experience TDI at least once [ 8 ]. Based on other epidemiologic studies, 50% of children have suffered from TDIs [ 9 , 11 ]. According to previous investigations, the prevalence of dental traumas is reported 4% -30% in developed countries [ 12 ]. This high prevalence of TDIs reveals a need for dental care programs including public and parental dental education [ 13 , 14 ]. Many studies have evaluated parental knowledge of TDIs. Majority of these studies revealed insufficient awareness in parents especially fathers, and therefore emphasized the need for further parental education [ 15 , 25 ]. However, there are only two comparable studies conducted in Iran. Navabazam et al. [ 15 ] observed that the prevalence and reasons of TDIs in schoolchildren of Yazd are similar to other countries, and Jabarifar et al. [ 16 ] reported that knowledge level of Iranian mothers of TDI management is relatively low. No reported Iranian study has assessed whether schoolchildren parents in Yazd (central Iran) are aware enough to manage TDI cases. Therefore, the aim of this study was to evaluate the knowledge of parents of school-aged children about dental trauma and to identify the effect of demographic variables such as age, gender, and education on their responses.

## Materials and Method

In this descriptive cross-sectional study, 300 elementary school children’s parents from Yazd (in Iran) were selected by cluster sampling. The written informed consent was obtained at enrollment, and the standardized questionnaire [ 16 ] was distributed among parents. The Institutional Review Board (IRB) of School of Dentistry, Shahid Sadoughi University of Medical Sciences approved the validity of the questionnaire. In order to confirm the reliability of the questionnaire, a pilot study was carried out on 30 subjects who were not included in the study. The data collected from the pilot study was analyzed by SPSS software. The reliability coefficient (Cronbach’s alpha) was 0.85, indicating an acceptable reliability. All the present parents at the time of the study were included. Parents who were reluctant to participate as well as incomplete questionnaires were excluded from the study. The questionnaire consisted of three parts. First part contained demographic questions including age, gender, job, education, number of children, and education (Table 1). In second part, five general questions were asked about previous experiences of traumas or related education (Table 2). The third part included 16 questions in two subparts on parents’ knowledge of TDIs. The first subpart consisted of four multiple choice questions about traumas to orofacial region, and the second subpart included 11 questions about a trauma case (a child who comes home with a tooth in his hand) (Table 3).

**Table 1:** Demographic characteristics of parents.

Demographic information	n(%)
**Gender**	
Female	223(75.3%)
Male	73(24.7%)
**Age**	
22-29	73(24.6%)
30-34	122(41.2%)
35-54	101(34.1%)
**Education**	
Not educated	34(11.5%)
Middle School	45(15.2%)
High school diploma	151(51%)
Bachelor’s degree or higher	66(22.3%)
**Occupation**	
Housewife	201(67.9%)
Clerk	33(11.1%)
Worker	24(8.1%)
Businessman	35(11.8%)
Retired	2(0.7%)
Unemployed	1(0.3%)

**Table 2:** Frequency distribution of answers to questions related to previous experiences of traumas or related education.

Question	n(%)	
A1- Have you ever observed a dental trauma?	Yes	198(66.9%)
	No	98(33.1%)
A2- Have you or your children ever had a traumatized tooth?	Yes	212(71.6%)
	No	84(28.4%)
A3- Have you ever got information about dental injury and its management?	Yes	140(47.3%)
	No	156(52.7%)
A4- If you have answered “yes” to the previous question, how did you get this information?	Television	60(20.3%)
	Book/Magazine	16(5.4%)
	Dentist	48(16.2%)
	Friends/Family	10(3.4%)
	Health centers	20(6.8%)
	Internet	3(1%)
A5-Are you interested in education about first aid measures related to oro-facial traumas?	Yes	65(22%)
	No	231(78%)

**Table 3:** Part 3- knowledge of parents.

Questions	n(%)	
Situation 1: You encounter a trauma to oro-facial region of your child…		
B1. Where is the first part that you check?	a.Head	154(52%)
	b.Face	48(16.2%)
	c.Mouth	44(14.9%)
	d.Teeth	26(8.8%)
	e. Would not know	24(8.1%)
B2. What is the first and best reaction when the face is traumatized?	a. To check the mouth and teeth	14(5%)
	b. To find the lost tooth/tooth fragment and replantation	123(43.8%)
	c. To take the child to a health center while the tooth is kept in water	108(38.4%)
	d. Other	13(4.4%)
	e. Would not know	41(13.9%)
B3. Which facial parts are most susceptible to trauma?	a. Lip	76(25.7%)
	b. Upper anterior teeth	171(57.8%)
	c. Lower anterior teeth	22(7.4%)
	d. Posterior teeth	1(0.3%)
	e. Would not know	26(8.8%)
B4. If the accident were in a dirty place, how would you decide about the tetanus vaccine?	a. Call a physician	45(15.2%)
	b. Call a dentist	17(5.7%)
	c. Go to a health center	217(73.3%)
	d. Other	3(1%)
	e. Would not know	14(4.7%)
Situation 2: Your child has come to home and a tooth is in his/her hand…		
C1. What do you do first?	a. Call a physician	8(2.7%)
	b. Call a dentist	43(14.5%)
	c.Take the child to hospital	198(66.9%)
	d.Other	17(5.7%)
	e. Would not know	30(10.1%)
C2. Can you recognize if the tooth is primary or permanent?	a. Yes	122(41.2%))
	b. No	174(58.8%)
C3. Do you think is it necessary to replant the tooth?	a. Surely	7(2.4%)
	b. Would not know	55(18.6%)
	c. No	189(63.9%)
	d. It depends on tooth.	45(15.2%)
C4. Can you replant the avulsed tooth?	a. Yes	8(2.7%)
	b. No	248(83.7%)
	c. Would not know	23(7.8%)
	d. It depends on situation.	17(5.7%)
C5. Do you think how much time you have to replant the tooth?	a. 10 minutes	14(4.7%)
	b. 15 minutes	13(4.4%)
	c. 20-30 minutes	20(6.8%)
	d. More than 20 minutes	8(2.7%)
	e. Would not know	241(81.4%)
C6. If you cannot replant the tooth, what are your reasons?	a. Lack of information	159(53.7%)
	b. Fear	28(9.5%)
	c. I do not know whether I can do it or not	89(30.1%)
	d. Other reasons	20(6.8%)
C7. If the avulsed tooth is contaminated, what should you do?	a. Gently scrub with a soft brush	19(6.4%)
	b. Replant the tooth without cleaning	2(0.7%)
	c. Rinsing with tap water	25(8.4%)
	d. Cleaning with moistened gauze/cotton ball	59(19.9%)
	e. Tooth is not replantable	59(19.9%)
	f. Other	12(4.1%)
	g. Would not know	120(40.5%)
C8. If the avulsed tooth is fractured, what should you do?	a. Replant the tooth anyway	5(1.7%)
	b. Call a dentist	218(73.6%)
	c. Would not know	63(21.3%)
	d. Other	10(3.4%)
C9. If you did not replant the tooth, how would you preserve it until you get to a dentist?	a. Ice	33(11.1%)
	b. Water	63(21.3%)
	c. Alcohol	9(3%)
	d. Milk	18(6.1%)
	e. Saliva	14(4.7%)
	f. Child’s hand	1(0.3%)
	g. Disinfectant solution	40(13.5%)
	h. other	4(1.4%)
	i. Would not know	114(38.5)
C10. What should you do if there were bleeding?	a. Stop bleeding by getting the child to bit on a handkerchief	169(57.1%)
	b. Take the child immediately to the dentist	94(31.8%)
	c.Wash the mouth with water	23(7.8%)
	d. Other	5(1.7%)
	e. Would not know	5(1.7%)
C11. Do you think you need more training in dental trauma management?	a. Yes	12(4.1%)
	b. No	259(87.5%)
	c. No idea	25(8.4%)

Lastly, parents were asked whether they needed more education on TDIs. In order to define the knowledge score, correct and incorrect answers got one and no point, respectively. Therefore, the score range was between 0 and 15. The scores higher than 10.5 was considered good, while scores between 7.5 and 10.5 and the scores lower than 7 indicated a medium and low level of knowledge, respectively. Data were analyzed by SPSS software version 11 (SPSS® Inc.) using T-test and ANOVA, and a P value of <0.05 was considered statistically significant.

## Results

Demographic characteristics of participants are shown in Table 1. A total of 296 parents participated in this study (223 mothers, 73 fathers). Mean age of participants was 33.8± QUOTE ± 5. About 75% of participants were mothers. The number of different answers to part 2 of questionnaire is shown in Table 2. Table 3 shows the responses to the third part of questionnaire, which evaluated the parents’ knowledge of TDI management (Tables 1-3). Despite the fact that most parents had previous self-experience (71%) or close observation (67%) of a TDI case, their knowledge about TDI management was inadequate (average score = 7.03). Only 41.2% of parents reported that they were able to distinguish between permanent and primary teeth. Moreover, only 6.4% claimed that they could replant an avulsed tooth. Knowledge of a proper storage media was also insufficient, since only 6.1% and 4.7% of parents chose milk and saliva as a storage medium, respectively. Whereas the majority of parents had not adequate knowledge about TDI, they were reluctant to learn about it and its management. According to T-test and ANOVA statistical tests, there was no significant relationship between knowledge and age (*p*= 0.155), gender of parents (*p*= 0.113), gender of children (*p*= 0.776), occupation (*p*= 0.112), and the information source of TDIs management (*p*= 0.160) (Tables 4-8). 

**Table 4:** Comparison of knowledge score of different age groups according to ANOVA test (*p*= 0.155).

Age (years)	Number	Mean knowledge score	Minimum score	Maximum Score
22-29	73	6.69	2	12
30-34	122	7.04	2	12
35-54	101	7.26	1	11
Total	296	7.03	1	12

**Table 5:** Comparison of knowledge scores regarding the gender of parents according to T-test (*p*= 0.113).

Gender	Number	Mean knowledge score	Maximum	Minimum
Female	223	6.93	12	2
Male	73	7.34	12	1
Total	296	7.03	12	1

**Table 6:** Comparison of knowledge scores regarding the gender of children according to T-test (*p*= 0.776).

Gender	Number	Mean knowledge score	Maximum	Minimum
Female	139	7.00	12	1
Male	157	7.06	12	3
Total	296	7.03	12	1

**Table 7:** Comparison of knowledge scores regarding the occupation of parents according to ANOVA (*p*= 0.112).

Occupation	Number	Mean knowledge score	Maximum	Minimum
Clerk	33	7.74	11	4
Worker	24	7.20	12	2
Businessman	35	6.90	12	2
Retired or unemployed	3	6.97	11	1
Housewife	201	6.90	12	2
Total	296	7.03	12	1

**Table 8:** Comparison of knowledge scores regarding the information source according to ANOVA (*p*=0.112).

Information Source	Number	Mean knowledge score	Maximum	Minimum
Television	60	7.21	12	2
Newsletter, Book and Brochure	16	8.37	11	6
Dentist	48	7.66	11	4
Friends	10	8.20	10	5
Health Centers	20	7.55	10	4
Internet	3	8.66	12	5
Total	157	7.60	12	2

However, the relationship between parents’ knowledge and parental educational level was statistically significant (*p*= 0.010), and least significant difference test (LSD) test showed that knowledge score of parents with Bachelor’s degree or higher educational levels (7.83±4) was significantly more than other parents (Table 9).

**Table 9:** Comparison of different educational levels regarding the knowledge scores according the ANOVA test (*p*= 0.010).

Educational level	Number	Mean knowledge score	Maximum	Minimum
Not educated	34	6.97	11	2
Middle school	45	6.66	11	3
High school diploma	151	6.80	12	1
Bachelor’s Degree or Higher	66	7.83	12	5
Total	296	7.03	12	1

## Discussion

TDIs are one of the most common dental accidents, which may occur in any situation.

While TDIs are not completely preventable, immediate reactions minimize further complications [ 1 , 2 ]. Based on the International Association of Dental Traumatology guideline, in cases of permanent tooth avulsion, which is a serious dental injury, the best treatment in the field of accident is immediate replantation of the tooth, and if not possible, the tooth should be saved in a suitable liquid medium as milk. It should be emphasized that the tooth should not be kept in water. In contrast, deciduous teeth should not be replanted. If the tooth is contaminated, it is suggested to rinse it gently under tap water before replantation [ 17 ]. 

In the present study, we observed that there was not a significant relationship between demographic factors including age, parents’ gender, occupation, child’s gender, and knowledge of parents in TDI management. There are limited similar studies conducted in this field; Andersson et al. [ 20 ] also showed that the age and gender of parents were not significantly related to their knowledge. However, the parental educational level was the only factor related to their TDI management knowledge, and this finding was compatible was those of Ozer et al. [ 26 ]. 

Jabarifar et al. [ 16 ] reported that 37% of mothers had experienced dental trauma in their children. Shashikiran et al. [ 27 ] showed that 47% of parents from urban areas and 42% of parents from rural areas of India had previous experiences of traumas to their children teeth. Therefore, based on previous studies, it seems that nearly half of parents especially mothers faced at least one TDIs to their children [ 15 , 26 ]. 

Similarly, our results revealed that 67% of parents had observed TDIs in their own childhood or their children and 71% of parents experienced TDIs whether in their children or themselves. This finding is indicative of two important points. First, it can be concluded that the prevalence of TDIs are noticeable in Yazd (central Iran). Second, the knowledge of these parents is expected to be higher than the parents who had not experienced such traumas. However, while the latter conclusion is confirmed in some studies [ 16 , 18 , 19 ], it was not observed in the present study.

One of the most important key points in management of TDIs in young children is the ability to discriminate the permanent teeth from primary teeth [ 17 ]. In the present study, only 41.2% of parents could discriminate between permanent and primary teeth, which shows the critical need for more education.

Regarding the avulsed teeth, only 2.4% of parents knew that the best management is replantation of the avulsed tooth, and surprisingly, more than 63% of parents thought that they should not replant the avulsed tooth. Other parents (18.6%) answered that they would not know how to manage this situation. More than 53% of parents stated that lack of knowledge is the main reason for not replanting the avulsed tooth. They mentioned that they do not know how to replant the tooth. However, in some parents (9.5%), fear was the main reason. Other parents (30%) did not know that replantation is possible at all. These findings are compatible with previous studies, which reported a low level of parental knowledge regarding replantation of avulsed tooth [ 16 , 20 , 22 , 26 , 27 ]. This lack of knowledge will inevitably lead to an inappropriate reaction and inability to manage these situations.

An appropriate storage medium is a medium able to save the vitality and adhesion of cells, and milk and the patient’s saliva would be first available choices in these situations [ 23 ]. In the present study, most parents (89.2%) had not proper knowledge about storage media. While only 6.1% and 4.7% of parents chose milk and saliva, respectively, 21% chose water as the best medium. Jabarifar et al. [ 16 ] reported that 34% of mothers in Isfahan (in Iran) correctly chose milk and saliva as the best storage media. This shows that the level of parental knowledge in Yazd is considerably lower than Isfahan, which is a more crowded and developed city in Iran. In the studies of Ozer et al. [ 26 ], Santos et al. [ 22 ], and Sanu et al. [ 19 ], less than 10% of parents had enough knowledge regarding the proper storage media.

Regarding the situations in which the avulsed tooth is contaminated, only 8.5% of participants chose “rinsing with tap water” which was the correct answer as the best way to clean the tooth, however, more than 40% stated that they do not have enough knowledge about cleaning the tooth. Other parents stated that the tooth is not replantable anymore (20%), or they would clean the tooth with a wet gauze or cotton ball (20%) or a soft brush (6.5%). Similarly, Ozer et al. [ 26 ] reported that only 5.9% of parents knew the best method to clean the avulsed tooth.

Surprisingly, while most parents did not have enough knowledge to manage TDIs, 85% of participants stated that they do not tend to be educated in this field. This finding should be further more analyzed in future studies and the underneath reasons are to be found out. In contrast, Ozer et al. [ 26 ] reported that 60% of parents who had not enough knowledge in TDIs management acknowledged the importance of being educated and 94% of all participants were interested in getting informed in this field.

In another study [ 28 ] conducted by the same researchers in Yazd, Iran, it was shown that knowledge of schoolteachers on emergency management of TDIs was also poor, and this confirms the absolute necessity of education of parents in TDI management.

It is strongly recommended to study the basic reasons of lack of awareness in parents in order to help them to improve their knowledge. In addition, educating parents on TDIs management and evaluating its effect can be a potential part of similar future studies.

## Conclusion

The present study revealed a considerable lack of knowledge of TDIs management in parents from Yazd, Iran, which influences their function, and subsequently the later complications of TDIs in their children. Therefore, there is an urgent need to improve the dental awareness of parents in this city.
